# Diclofenac patches for postoperative shoulder pain

**DOI:** 10.4103/0973-6042.41035

**Published:** 2008

**Authors:** Lennard Funk, Rowena Umaar, Adeyinka Molajo

**Affiliations:** Bridgewater Hospital, 120 Princess Road, Manchester, M15 5AT, United Kingdom

Sir,

Major shoulder surgery can now be performed as day case procedures, thanks to advancements in minimally invasive techniques and regional anesthesia. However, once the regional anesthetic block has worn off, at an average of 20h, the rebound pain can be significant. Many day-case patients are at home at this time, without immediate access to stronger analgesia. Although patients are advised to preload with anti-inflammatories and analgesics prior to the regional block wearing off this does not always occur due to variability in time of regional wearing off and patient compliance.

Diclofenac hydroxyethylpyrollidine (DHEP) patches have proven to reduce pain and inflammation in the direct treatment of acute and chronic inflammatory conditions.[1–11] They release the active ingredient over a 12-24 hour period,[13] with less systemic side-effects than oral forms and better patient compliance.[14] DHEP patches have also proven beneficial for day-case laparoscopic surgery by reducing postoperative analgesic requirements and hospital stay.[15]

We assessed the analgesic effect and postoperative recovery of diclofenac patches compared to tablets in day case arthroscopic shoulder surgery. Thirty one patients underwent arthroscopic shoulder procedures over a six-month period in a specialist day surgery hospital. The patients were randomized in two groups: Group 1 (17 patients) received diclofenac tablets in addition to paracetamol and codeine and Group 2 (14 patients) had diclofenac patches in addition to paracetamol and codeine. All patients also had a single shot interscalene regional with levobupivicaine, intravenous paracetamol intraoperatively and cold compression therapy. The patches were changed twelve hourly after application and continued to be used at least 48h postsurgery. Simple randomisation was applied and ethical approval not specifically sought, although all patients were consented accordingly. The patients and investigators were not blinded.

[Table 1] lists the procedures performed. The most common was arthroscopic shoulder stabilisation procedure.

**Table 1:** Types of shoulder procedures performed and number.

Procedures	Number
Arthroscopic Shoulder Stabilisation	17
Arthroscopic SLAP Repair	1
Arthroscopic Subacromial Decompression	3
Arthroscopic AC Joint Excision	1
Arthroscopic Rotator Cuff Repair	4
Arthroscopic Capsular Release	3
Other	2

All patients were contacted by telephone an average of 48h postsurgery and completed a 5 point visual analogue pain scale, where 0 was no pain and 5 maximum pain. They were also asked about any additional analgesic requirements and their comfort level. *Chi-squared* statistical analysis was performed using ‘Analyse-it’ software package.

**Figure 1:**
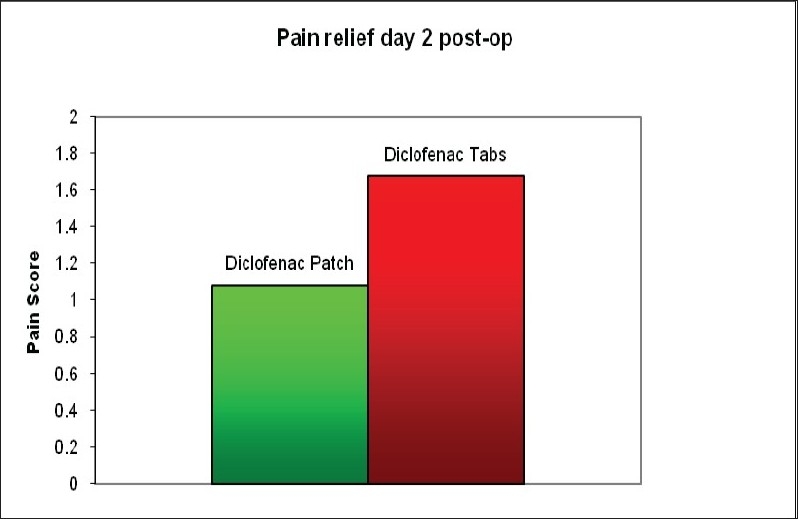
Post-operative pain score on the second postoperative day

The mean age of Group 1 was 32.7 years and Group 2 was 40.5 years (p>0.05). The male:female ratio in group 1 was 14:3 and in group 2 was 12:2. The mean surgical time was 75 minutes in Group 1 and 72 minutes in Group 2 (p>0.05). The mean time from end of procedure to discharge from the day surgery hospital was three hours and 43 minutes for Group 1 and three hours and 31 minutes for Group 2 (p>0.05). The mean pain score for at 48h post-op was 1.7 in Group 1 and 1.1 in Group 2 [Figure 1]. This was significant (p=0.031). One patient in each group required additional analgesia in the form of Tramadol. There were no side-effects to the diclofenac tablets or patches.

In conclusion, diclofenac patches provides significantly better pain relief compared to tablets in the early postoperative period following arthroscopic shoulder surgery.
